# Research on Terminal Access Performance of Beam-Hopping Satellite in IoT Scenario

**DOI:** 10.3390/s23031428

**Published:** 2023-01-27

**Authors:** Yulei Nie, Gengxin Zhang

**Affiliations:** 1 College of Integrated Circuit Science and Engineering, Nanjing University of Posts and Telecommunications, Nanjing 210023, China; 2 School of Communications and Information Engineering, Nanjing University of Posts and Telecommunications, Nanjing 210023, China

**Keywords:** LEO, sIoT, terminal access, collision, R-SSA

## Abstract

In recent years, low-orbit satellites have become an important development direction in satellite IoT systems. The number of terminals is large and data collisions occur frequently in the low-orbit satellite IoT scenario. How to design a reliable random access protocol to improve the tolerance of the system for collision is one of the research hotspots in this field. In this paper, the random access protocol, used in the Internet of Things (IoT), for low-orbit satellites is studied, and the access process of the IoT terminals in the scenario is constructed. The access performance of the SSA protocol is analyzed and an improved SSA random access strategy, called Retransmission-SSA (R-SSA), is proposed. The simulation results show that the designed R-SSA can effectively tolerate the signal conflicts between terminals in the beam-hopping LEO IoT scenario and improve the probability of the concurrent access of low-orbit sIoT terminals.

## 1. Introduction

Satellite Internet Constellation refers to the newly developed giant communication satellite constellation that can provide data services and realize Internet transmission functions.

In China, for example, terrestrial communication networks cover only approximately 20% of its land area [1]. The low-orbit satellite Internet of Things (IoT) can be used as a supplement and extension of the ground IoT and effectively overcome the problem of insufficient coverage capacity of the ground IoT in oceans, mountains, deserts, and other areas in cases of seamless global coverage. In the event of natural disasters and emergencies on the ground, satellite IoT can still work normally, and it is also easy to provide uninterrupted network connectivity to large-scale moving targets (aircraft, ships, etc.). The IoT of low-orbit satellites is a major national demand and has a wide range of application prospects in the field of the national economy and national defense [2]. Therefore, the IoT of low-orbit satellites has received extensive attention around the world and is also a hot spot in the current research.

At present, the commonly used transmission mechanisms of the terrestrial IoT include NB-IoT and LoRa. However, both of these are designed for terrestrial cellular systems, and the data transmission characteristics are different from those of the satellite IoT scenarios [3]. It should be noted that the use of low-orbit satellites to implement the IoT is very different from the conventional ground IoT. First of all, the coverage of a single satellite reaches the order of thousands of kilometers, far exceeding the kilometer order of the coverage area of ground base stations. At the same time, although the number of terminals served by a single satellite is huge, the connection density supported per unit area is much lower than that of ground base stations, which is mainly suitable for application scenarios with low connection density in wide areas. On the other hand, because low-orbit satellites are in a high-speed motion state, the rapid movement of satellite beams will bring a large Doppler shift, and the network connection relationship between the terminal and the number of service terminals will also change rapidly and dynamically. The distance of the communication between satellite IoT terminals is also much higher than that between traditional ground IoT terminals, and the improvement of propagation loss and delay is not conducive to the design of terminal low power consumption and communication protocols. Finally, the frequency, power resources and processing capacity of satellites are limited by factors such as size, weight and energy supply, which makes it impossible to install large-aperture antennas and support complex onboard processing missions.

At present, there are dozens of low-orbit satellite communication systems built, under construction or planned to be built, around the world, most of which can provide IoT services. Take the Orbcomm system, for example, which is specifically designed for bidirectional short data transmission [4]. It provides functions including remote data collection, system monitoring, tracking and positioning of vehicles, ships and mobile facilities, the transmission of short messages, sending and receiving e-mails, etc. Another well-known satellite IoT system is the OneWeb system, which currently has 110 satellites in orbit [5]. The system plans to deploy a total of 48,000 satellites, with the initial phase planned to launch 648, at an orbital altitude of approximately 1200 km. OneWeb can provide services such as oil and gas pipeline monitoring and the Internet of Vehicles. At present, the world’s most well-known low-orbit satellite IoT system is the “Starlink” system; since Elon Musk proposed the system construction plan in 2015, 3399 satellites have been launched, 3168 of which are in orbit (2700 are in service) [6]. Starlink plans to deploy a total of 42,000 low-orbit satellites in the future, and the existing satellites in orbit can already provide high-throughput, low-latency satellite Internet services to global users, which can be divided into four categories: home, commercial, RV, and marine.

In terms of low-orbit satellite IoT architecture, the five-layer architecture of the spatial information network that integrates Geostationary Orbit (GEO), Medium Earth Orbit (MEO), Low Earth Orbit (LEO), High Altitude Platform Station (HAPS) and the ground network is the mainstream design idea. With the research results of the terrestrial 5G network and advanced technology, the establishment of a satellite/HAPS/ground integrated information network structure [7] can provide global users with broadband access, mobile communication, navigation, and positioning services. Some researchers separate business and control information based on the protocol architecture of software-defined networks [8] and find frequency resources through spectrum sensing and other technologies in order to improve the network service quality.

In terms of terminal access, the low-orbit satellite IoT system includes a variety of terminals, with a variety of services, and a large number of terminals use the method of randomly sending data, resulting in data conflicts and increasing latency. The above situation means that the low-orbit satellite IoT need to carefully consider the design and selection of multiple access schemes. The multi-access scheme of a low-orbit satellite IoT system needs to provide a high transmission rate for massive access terminals, and at the same time, it needs to have a high degree of adaptivity, in order to flexibly adapt to the dynamic changes of low-orbit satellite network topology. On the other hand, it is difficult for traditional single-beam satellite communication systems to meet the access requirements of massive terminals due to time-frequency resource limitations, the particularity of the services, and the low coverage of low-orbit satellites to IoT systems. The use of a multi-beam satellite communication system to multiplex time-frequency resources to improve the system terminal capacity, and the use of beam-hopping for the on-demand scheduling of resources, are the main means and research hotspots to solve the above problems in recent years.

The main contents of this paper are arranged as follows: the second section compares and analyzes the commonly used multiple access methods, and analyzes the shortcomings, focusing on the Contention Resolution Diversity Slotted ALOHA(CRDSA) and Spectrum Spread ALOHA (SSA) suitable for dealing with multiple conflict scenarios. The third section establishes the low-orbit satellite IoT terminal beam-hopping management scenario and simulates and analyzes the access mode performance of the SSA in the established scenario. Then, the R-SSA access method combined with the idea of collision retreat is proposed, and simulation analysis is carried out. Finally, the paper summarizes and discusses the next research direction of the method.

## 2. Analysis of Access Mode of Low-Orbit Satellite IoT Scenarios

The current research on the satellite IoT random access protocol is mainly aimed at the uplink access scenario of devices within the coverage range of a single satellite beam. The earliest ALOHA protocol was first proposed by the University of Hawaii in 1968, and after decades of development, there are now a variety of ALOHA multiple access protocols for satellite communications and terrestrial communications. These include Pure ALOHA, Spread Spectrum ALOHA, Slotted ALOHA, Reservation ALOHA, and so on.

### 2.1. Traditional ALOHA Protocol

#### 2.1.1. Slotted ALOHA

To improve the throughput rate of the pure ALOHA, Roberts proposed the Slotted ALOHA protocol in 1972, which reduces the probability of packet collision by narrowing the vulnerable interval of the pure ALOHA. The Slotted ALOHA was the first proposed satellite random access protocol [9], and the Slotted ALOHA doubles the maximum throughput of the system compared to the pure ALOHA.

In the Slotted ALOHA, the timeline is divided into several packet transmission time slots, with a width equal to 1, and the data of the user terminal are stored in the buffer when the packet arrives, before being sent, until the next time slot starts. Obviously, when only one packet arrives in a one-time slot, this packet can be sent successfully in the next time slot. When there are two or more user terminal data packets in a time slot, a collision will occur in the next time slot. Immediately after the user detects the feedback message that it senses a packet collision, an integer ε in the interval [1, *k*] is randomly generated with equal probability. From the beginning of the next time slot, after the εT delay, the collision packet is resent until the packet is successfully received by the satellite. The slotted ALOHA is more efficient in the random access scenario of short packets, but for multiple access communication with long data information and burst transmission, the Reservation ALOHA method is usually used. SA has the advantages of simple processing and high energy efficiency on the transceiver side, but its peak throughput is only 0.368 [10].

#### 2.1.2. Reservation ALOHA

When the user terminal needs to send long packets, the Reservation ALOHA method is often used. This method requires an appointment with the satellite first, and the sending time slot is allocated by the satellite. This mode avoids collisions and greatly improves the channel throughput compared to the pure ALOHA and slotted ALOHA. However, the waste of resources in the short message scenario of the scheduled ALOHA is serious, particularly when the actual business data are less than the reservation data, and the resource utilization rate is extremely low. Therefore, in the actual satellite IoT scenario, the complementary methods of the time slot ALOHA and reservation ALOHA are often used to deal with two business scenarios: long and short messages [11,12].

### 2.2. Improved ALOHA Protocol

#### 2.2.1. Contention Resolution Diversity Slotted ALOHA (CRDSA)

In 2007, Casini proposed CRDSA to improve the access efficiency of the time slot ALOHA [13]. In this scheme, the user randomly selects multiple time slots to send packets, and the receiving end uses the serial interference cancellation algorithm (SIC) to demodulate the collided packets in turn and uses the successfully demodulated packets to decode other colliding packets.

Specifically, CRDSA uses pointers to tell the slot location of the two copies of the packet based on multi-copy packet sending. The receiving node uses a serial interference cancellation algorithm to unpack the packet. The unpacking process starts with conflict-free copies of the packets, which can be recovered once they are detected. Then, according to the pointer information of the packet, another copy is found, and then the collision effect of these copies on the other packet copies is eliminated through signal processing. At this point, an iterative process of interference elimination has been completed. After the interference elimination is complete, some new packet copies may be restored, and a second interference cancellation process can then be launched based on these packet copies. By continuously iterating this interference elimination process, the receiver can recover as many packets as possible from the received signal.

**Figure 1:**
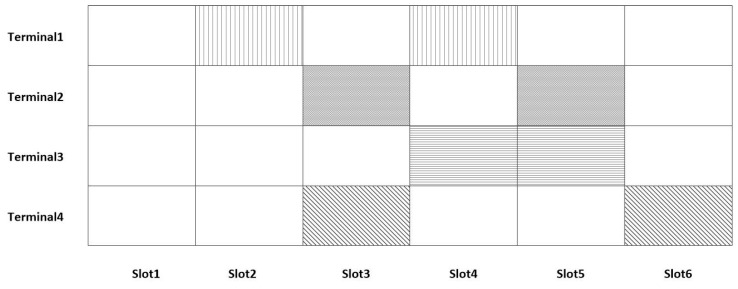
CRDSA access model.

As shown in Figure 1, the data collision model of the CRDSA data receive frame can be divided into three states for each time slot:

1. Idle state: no terminal devices are transmitting data during this time slot.
2. Ideal state: this time slot receives data from the terminal and only one terminal occupies this time slot; there is no conflict problem.
3. Overload state: there are terminals conducting data transmission in this time slot, but more than one terminal sends a copy of the packet at this slot, and there is a packet collision.

**Figure 2:**
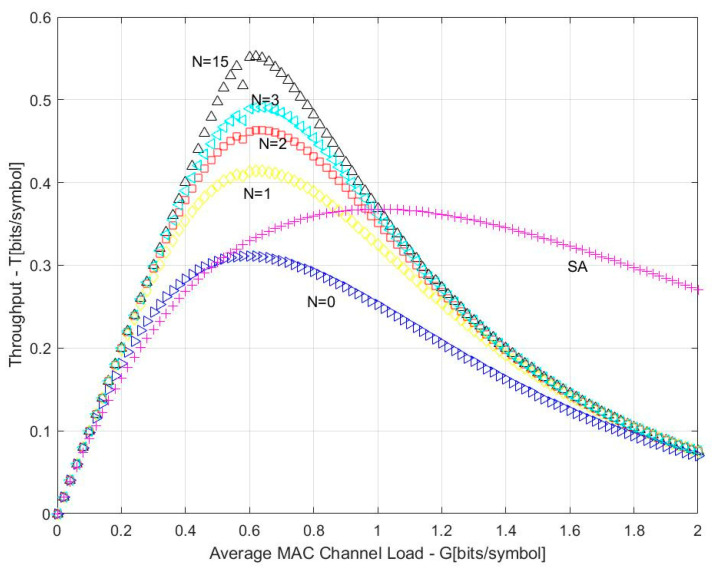
Performance of CRDSA.

In CRDSA, if all copies of a packet conflict, the packet cannot be recovered and can only wait for retransmission. As long as one copy of a packet is decoded, the other copy that conflicts with other packets can also be located and eliminate interference with other packets. As can be seen from Figure 1, one copy of the terminal 1 packet conflicts with copies of other packets, and the other packet copy is in the ideal time slot, so the packet copy of Terminal 1 can be recovered. Terminal 1, Terminal 3, and Terminal 4 are in the same conflict state, and only one copy of the packet has collided, so the packet copy of Terminal 3 and Terminal 4 can also be recovered. Both copies of the packet of Terminal 2 interfere with the copies of the other packets, and the packet copy of Terminal 2 cannot be recovered during the first packet recovery. However, as the packet copies of Terminal 1, Terminal 3, and Terminal 4 have been restored, the packet of Terminal 2 can also be recovered after a disturbance elimination process. Under different maximum iterations N, the access performance of CRDSA is as shown in Figure 2, and the main parameters used are shown in Table 1:

On the basis of CRDSA, Liva further reduces the probability of a packet collision by further optimizing the probability distribution function of the number of packets transmitted by the device, and proposes the Irregular Repetition Slotted ALOHA (IRSA) to further improve the system performance [14,15]. The CRDSA and IRSA protocols have been used in second-generation digital video broadcasting satellite systems [16]. Further improvements have been made for the CRDSA protocol, such as the SW-CRDSA Sliding Window based CRDSA (SW-CRDSA) [17], the Multi-Frequency CRDSA (MF-CRDSA) [18], etc.

#### 2.2.2. Spectrum Spread ALOHA

The spread spectrum ALOHA (SSA) protocol adds a spread spectrum modulation to the traditional ALOHA protocol [19]. SSA uses the strong autocorrelation characteristics of the spread spectrum sequences to realize the separation of signal collision users based on chip spacing [20]. When the collision signal delay exceeds one chip, two different user signals can be separated. As with the near-far effects in terrestrial 3G mobile communication systems, the SSA possesses significant performance degradation in the case of power imbalance. E-SSA is an enhanced version of SSA, which draws on the idea of CDRSA iterative reception [21], combines nonsynchronous spread spectrum technology with FEC and SIC on the basis of SSA, and realizes the separation of collision user signals through SIC, so that the algorithm approaches the single-user channel capacity at a low packet loss rate. The E-SSA scheme is robust to power imbalance, and all packets will be received correctly until the system load reaches a critical value by SIC technology [22]. Under the beam hopping satellite communication system with evenly distributed IoT users, SSA technology can be used to solve the problem of the simultaneous access of massive users and improve the collision tolerance performance of the system [23].

## 3. Analysis of SSA Access Performance in the Scenario of Beam Hopping Low-Orbit sIoT Scenario

The application of beam-hopping technology improves the on-demand service capability of satellite communication systems, gives full play to the large coverage advantages of satellite communication systems in satellite IoT scenarios, and maximizes the satellite communication capabilities in the emergency information transmission scenarios of wide-area distributed IoT devices. In this section, a beam-hopping service management and control scenario of the low-orbit satellite IoT communication system is constructed, and the collision number and throughput simulation using the SSA access mode are simulated in this scenario.

### 3.1. Architecture of Low-Orbit Satellite IoT

**Figure 3:**
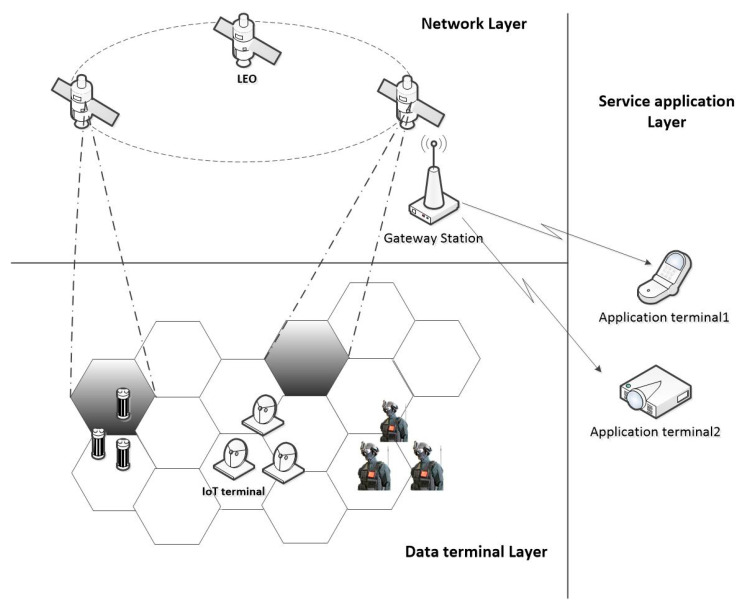
The architecture of Low-orbit satellite IoT with beam-hopping.

The use of low-orbit satellites to realize the IoT has a small propagation delay and transmission loss, while greatly improving the coverage of the IoT, and the use of beam-hopping technology to provide services for specific IoT terminals that can further improve the on-demand service capabilities of the system. The architecture of the IoT of low-orbit satellites is shown in Figure 3.

The low-orbit satellite IoT system depicted in Figure 2 is divided into the network layer, data terminal layer and service application layer. Among them, the network layer is mainly divided into two parts: the low-orbit satellite access network and ground access network. The terrestrial access network section is not discussed in this article. The data terminal layer is for various types of IoT devices. The service application layer provides users with application services based on the backhaul data of IoT devices.

There are a large number of uniformly distributed acquisition data terminals at the data terminal layer. Among them, the terminal that wakes up under special conditions will randomly generate data, sending requirements such as environmental monitoring terminals. Such terminals tend to be massive and widely deployed and the probability of data collisions is also higher. On the other hand, in emergency scenarios, such as rescue and disaster relief, IoT terminals in a certain area will have frequent data transmission needs. At this point, the required resources will increase dramatically. The application of beam-hopping technology in low-orbit satellite IoT systems can achieve flexible beam scheduling and resources are allocated on demand while meeting the data access of massive collection IoT terminals during normal system operation in emergency scenarios. Meanwhile, the SSA random access protocol is used in the low-orbit satellite communication system, which can effectively solve the access problem of massive terminal data. This paper focuses on the access performance of users using the SSA random access protocol under the evenly distributed beam-hopping satellite communication system of IoT terminals.

### 3.2. Access Process of Beam-Hopping Low-Orbit Satellite IoT System Based on SSA

In the scenario of the uniform distribution of IoT data terminals, satellite IoT terminals use the SSA random access protocol to access satellites. The IoT terminal synchronizes the time of the whole network through the timing system and determines the cell arrival time according to the obtained beam-hopping pattern information. Then the terminal accesses and transmits the data according to the sending needs, and the specific process is as follows:

1. Step 1: The terminal synchronizes the system time and obtains the beam-hopping pattern information.
2. Step 2: Determine the time when the beam reaches its own cell.
3. Step 3: Encode and frame the service data during the beam-hopping period.
4. Step 4: Spread spectrum using a predetermined PN sequence.
5. Step 5: According to the beam arrival time at the cell, calculate the time range where the head chip is located.
6. Step 6: In the time range where the header chip is located, randomly select a sending time t.
7. Step 7: Wait for the beam to arrive.
8. Step 8: Modulation, frequency conversion and amplification processing.
9. Step 9: Send information to the satellite at regular intervals according to the sending time t.

**Figure 4:**
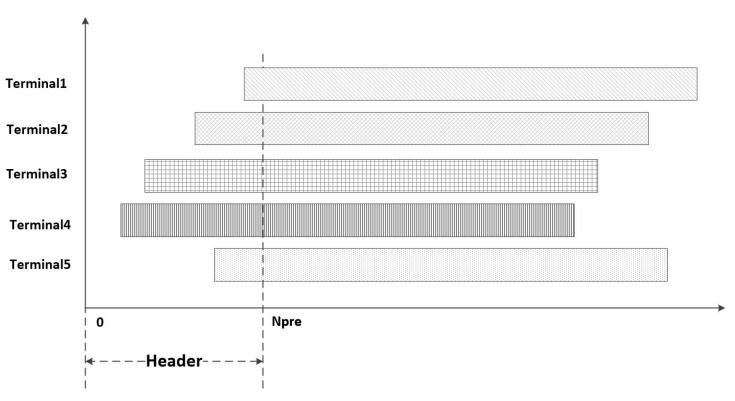
SSA access model.

As shown in Figure 4, during a beam-hopping period, all five terminals in the berth need to send data. Set the beam arrival time as $T_{0}$ and the header is set to 0 to $N{\;}_{pre}$ chip duration. Suppose the chip time is $T_{chip}$. Then the time of header is:

(1) $$T_{Header}\in \left[0,N_{pre}\times T_{chip}\right]$$

Each user selects the send time on the chip time from 0 to $T_{Header}$. Two users can recover their respective information at the receiving end by two or more pieces of transmission time apart when they use different PN sequences for spreading.

### 3.3. Collision and Throughput Performance in Low-Orbit Satellite IoT Beam-Hopping Scenario with SSA Protocol

Aiming at the low-orbit satellite IoT beam-hopping scenario, this paper uses SSA to evaluate the collision probability and capacity of the system under different demodulation capabilities.

The data grouping in the system arrives according to the Poisson process and the retransmission process after the emergence of grouping conflicts is still the Poisson process.

Under ideal discrete memoryless binary channel conditions, data conflicts are a major factor affecting the accuracy of packet transmissions. Let the length of each packet in the system be fixed-length $T_{0},$ which consists of L bits. Let the conflict interval for each bit in the grouping be δ chips. System groupings of length L bits will have L discrete collision windows and each collision window has a length of $\delta T_{c}$. The total conflict window is:

(2) $$W_{total}=L\times \delta \times T_{c}=L\delta T_{0}$$

According to the description of the system model, the service arrival probability obeys the Poisson distribution with parameter λ within one packet transmission time τ. Thus, the probability of the K packets arriving simultaneously during the τ time period can be expressed as follow:

(3) $$f\left(k;\lambda \tau \right)=\frac{{\left(\lambda \tau \right)}^{k}exp\left(-\lambda \tau \right)}{k!}$$

As each packet length is $T_{0}$, the time range for collisions between packets is $2T_{0}$. The header data sent before and during this window will cause collisions when there is a user sending data within the same window. The simulation parameters used are shown in Table 2.

**Figure 5:**
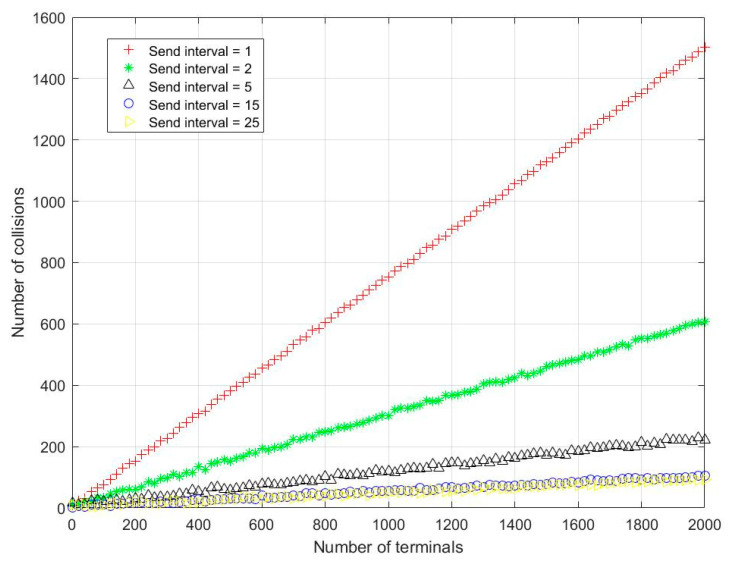
Number of collisions under different numbers of terminals in LEO satellite beam-hopping IoT access scenario.

The simulation results are as follows:

Figure 5 depicts the number of collision users for different system users. As can be seen from the figure, as the average sending time of system users increases, so does the number of user-sending data in the same collision window.

Figure 5 shows the probability that the number of users in the window where user collisions occur is greater than the demodulate threshold, and the normal transmission and reception cannot be completed when the parallel demodulation threshold is 10; that is, the true collision probability of the system.

**Figure 6:**
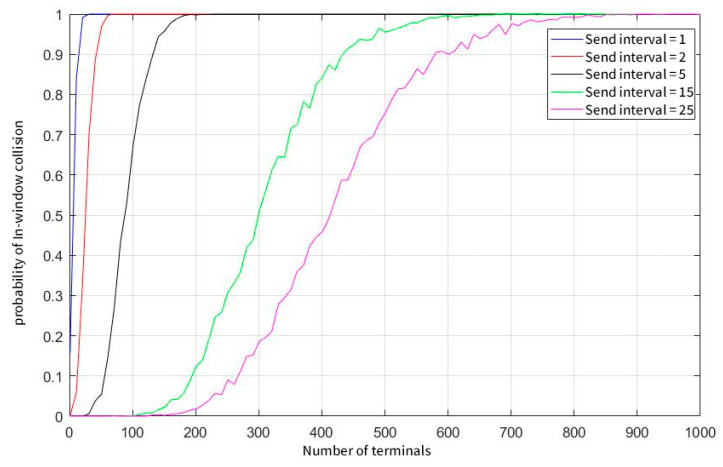
The probability of a window collision at concurrent demodulation thresholds equals 10.

As can be seen from Figure 6, as the number of system users increases, the probability of data appearing in the current window increases, resulting in an increase in the probability of collision. In addition, the user’s sending frequency also affects the probability that the window cannot be demodulated. When the number of system users reaches 900, even if a single user sends data with a low probability of sending every 25 s, there is a near 100% probability of colliding with the data of at least nine other users in the same window.

**Figure 7:**
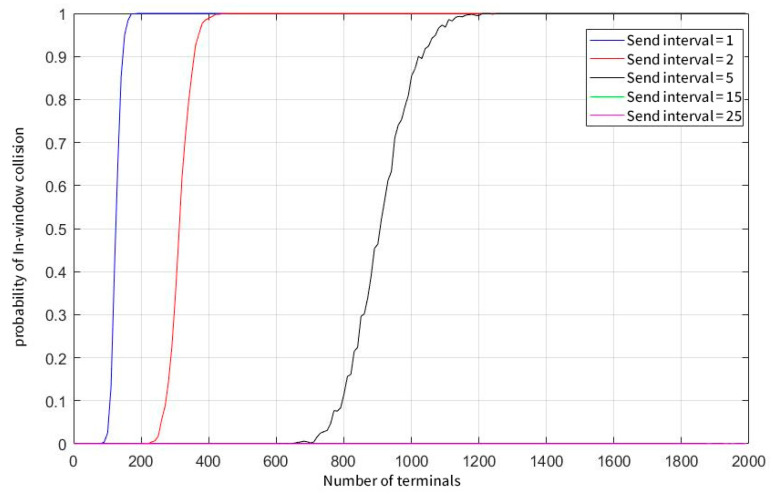
The probability of a window collision at concurrent demodulation thresholds equals 100.

Figure 7 shows that when the parallel demodulation threshold is raised to 100, the user capacity of the system is greatly improved. In a system of 2000 total users, regardless of whether the user sends data at a frequency of 15 s or 25 s, there is a high probability that no real collision will occur. When the user sends data at a frequency of once per second, the limited user capacity of the system is also increased by nearly 20 times.

**Figure 8:**
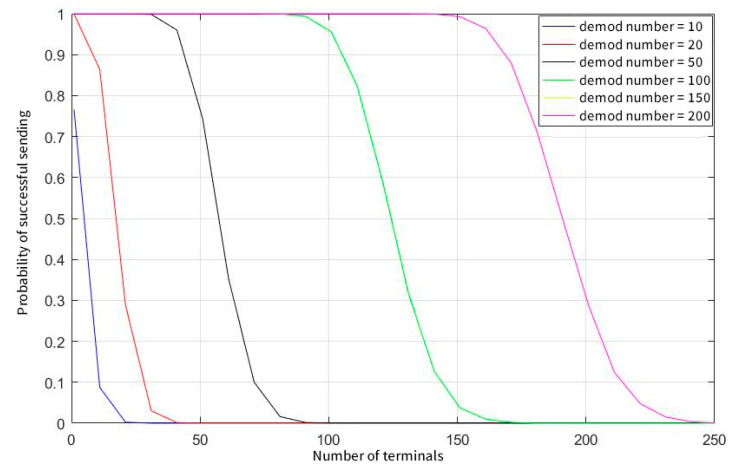
Probability of successful sending when sending interval of terminals equals 1.

**Figure 9:**
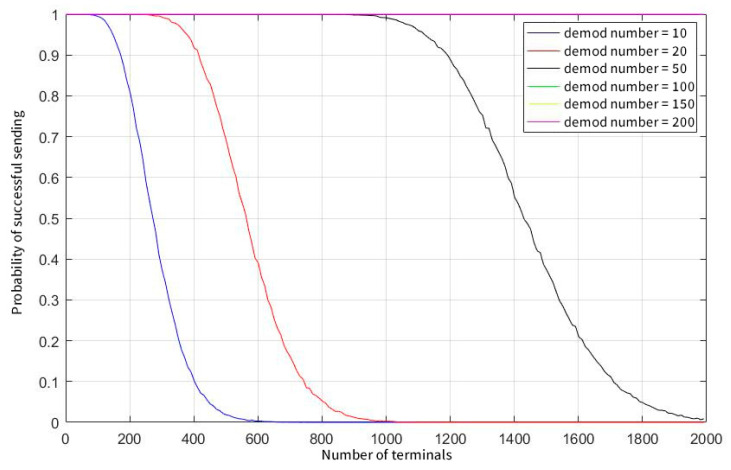
Probability of successful sending when sending interval of terminals equals 15.

Figure 8 shows that when a user sends data every second, the transmission success rate drops sharply as the number of users increases, and when the parallel demodulation threshold is set to 200, the peak capacity of the system is only 250. Figure 9 illustrates that when the user’s sending probability is further reduced, the capacity of the system is greatly increased again after the user sends data once in 15 s. In this case, a concurrent demodulation number of 50 can also guarantee the conflict-free sending and receiving of nearly 1000 users, and the limit capacity is close to the maximum number of users in the system.

**Figure 10:**
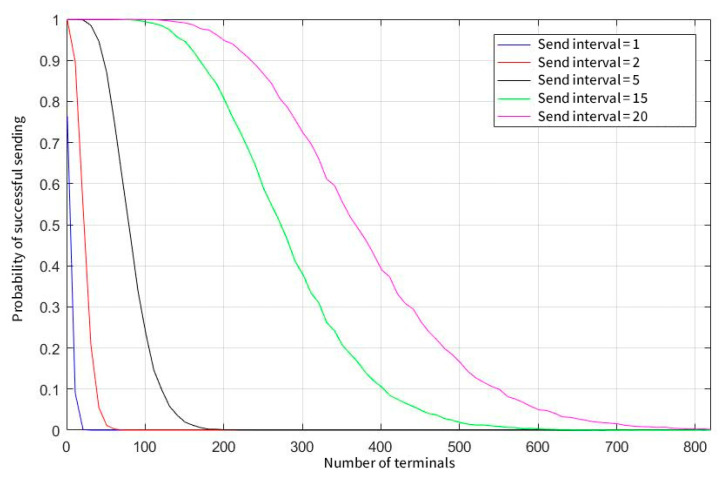
Statistics of successful sending probability at different sending intervals (concurrent demodulation thresholds equal to 10).

Figure 10 shows the effect of the different user-sending frequencies on the probability of successful transmission when the demodulation threshold is 10. As can be seen from the figure, the probability of successful transmission is significantly better than when the transmission frequency is low. On the other hand, as the number of users of the system increases, the probability of sending decreases sharply. In the case of the user sending data every 5 s, the system limit capacity is approximately 200, and if the user sends data every 25 s, the system limit capacity is approximately 800.

**Figure 11:**
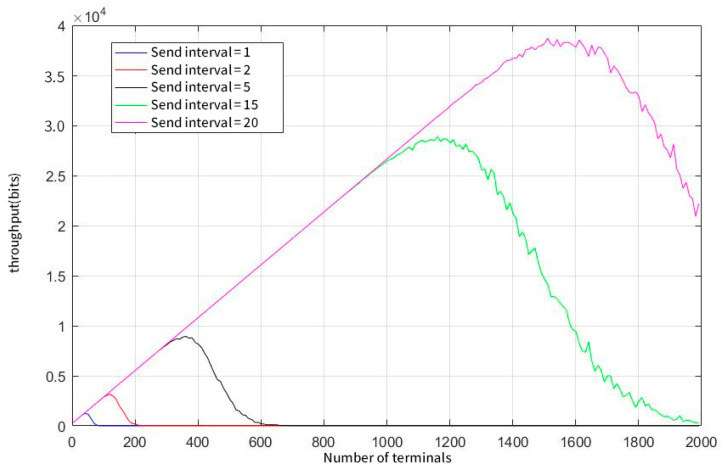
System throughput at a concurrent demodulation threshold of 50.

**Figure 12:**
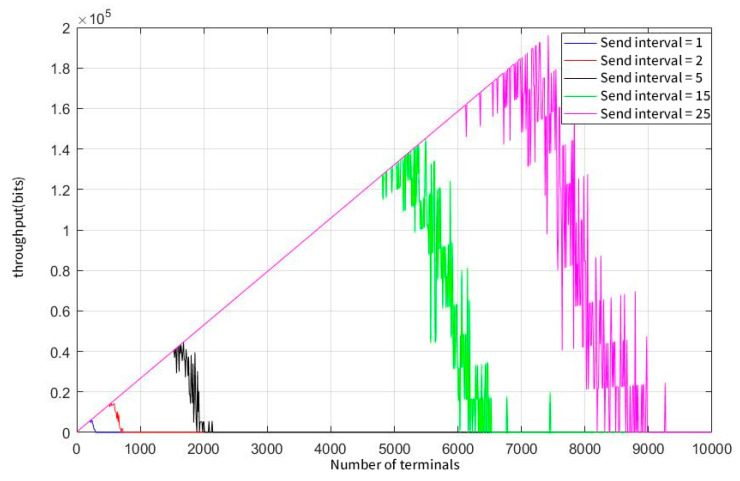
System throughput at a concurrent demodulation threshold of 200.

As can be seen from Figure 11, the reduction in the user sending frequency can greatly improve the effective throughput of the network, which is about 16 Kb with a demodulation capability of 50. Figure 12 illustrates that the system has exceeded the expected user number that the system required when the parallel demodulation threshold reaches 200. The peak was reached when the number of users was approximately 7,500. At this time, the effective throughput of the system is about 200 Kb.

### 3.4. Retransmission-SSA

#### 3.4.1. Collision Fallback Process

**Figure 13:**
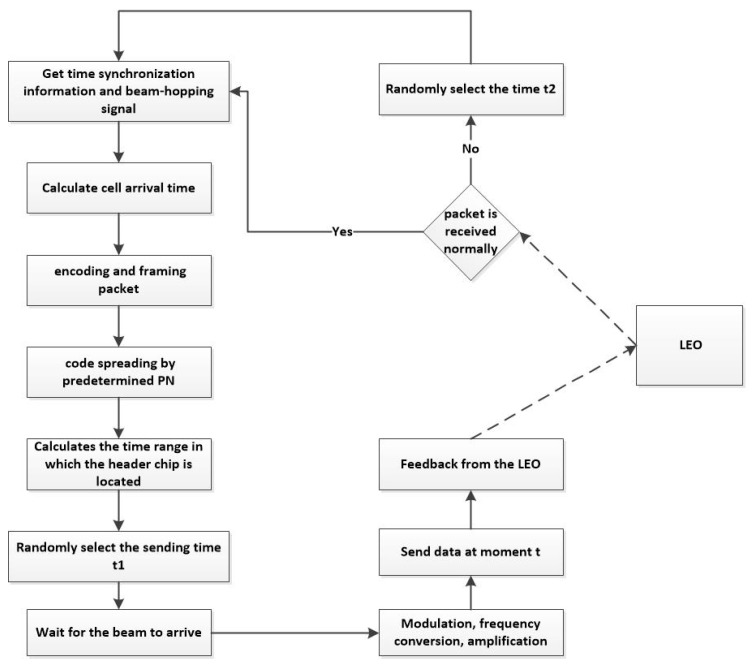
R-SSA access process.

As can be seen from the previous section, in the low-orbit satellite IoT scenario, the collision problem caused by massive random access can be alleviated by skipping the beam design and the SSA scheme. However, after a collision occurs, there is no mechanism to confirm whether the receiver received the information correctly, which will lead to packet loss. To further improve the system’s tolerance for collisions, this paper draws on the design concept of IRSA and improves the existing SSA protocol, which can be called Retransmission-SSA(R-SSA). By adding the collision fallback process, after the terminal sends data, the feedback information is used to start the random time retransmission timing, and the data will be retransmitted at a future time. The specific process is shown in Figure 13.

According to the description of the system model, the service arrival probability obeys the Poisson distribution, with parameter λ within one packet transmission time τ, as described in Section 3.3. The probability of n packets arriving at the same time is f(n;λΔt); where Δt is the maximum time difference between the signal reaching the different receiving nodes in each receive window and Δt=Nτ,N>1. Among these n packets, the probability of a collision between the m data and a particular packet follows a binomial distribution. The expression formula is as follows.

(4) $$P_{loop}^{n}\left(l:n,p\right)=\left(\begin{array}{c} n-1\\ m \end{array}\right)\cdot p^{m}\cdot {\left(1-p\right)}^{n-m-1}$$

In the formula, p is the collision probability. According to the nature of the Poisson distribution, the packets within the [t,t+Nτ] time slot will be evenly distributed when there are enough packets in the same window. Assuming that a packet arrives at moment $t_{0}$, the probability of collision can be obtained by the full probability formula due to the randomness of the backhaul retransmission, where $p_{1}$, $p_{2}$, and $p_{3}$ represent the collision probability under different circumstances.

(5) $$\{\begin{array}{c} p_{1}=\int_{t}^{t+\tau }\frac{t_{0}-t+\tau }{N\tau }dt_{0},\;\left(t\leq t_{0}<t+\tau \right)\\ p_{2}=\frac{2\tau }{N\tau },\;\left(t+\tau \leq t_{0}<N\tau -\tau \right)\;\\ p_{3\;=\;}p_{1},\;\left(N\tau -\tau \leq t_{0}<N\tau \right) \end{array}$$

#### 3.4.2. Simulation

In the low-orbit satellite beam-hopping IoT scenario, the designed SSA scheme with a collision fallback process is simulated, and the parameters used are shown in Table 3:

If the number of collisions at that moment is less than the demodulate threshold M, the collision is considered to not affect the demodulation, and vice versa, the true number of collisions is recorded.

**Figure 14:**
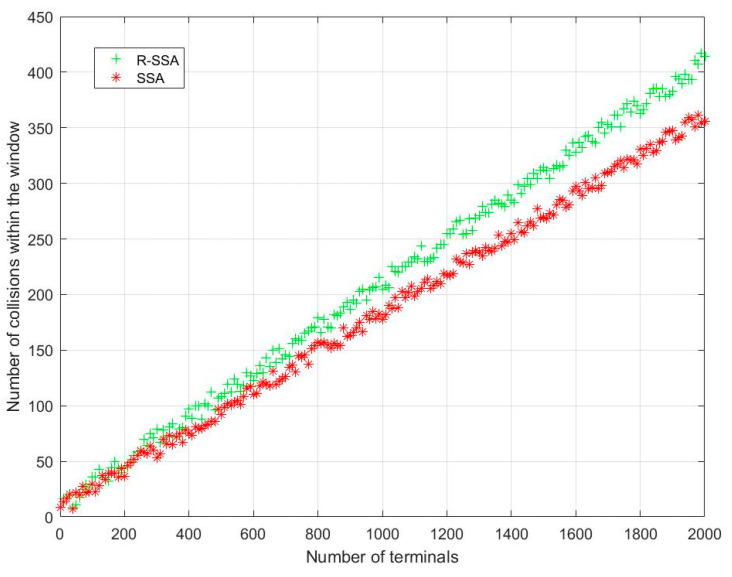
R-SSA vs. SSA in the number of collisions in the same collision window.

It can be seen from Figure 14 that the number of terminals sending data in the same collision window increases after the collision backoff mechanism is added, which means that the collision backoff mechanism increases the frequency of the terminal transmission. When the expected number of users is 400, there are nearly 420 terminals waiting to be accessed.

**Figure 15:**
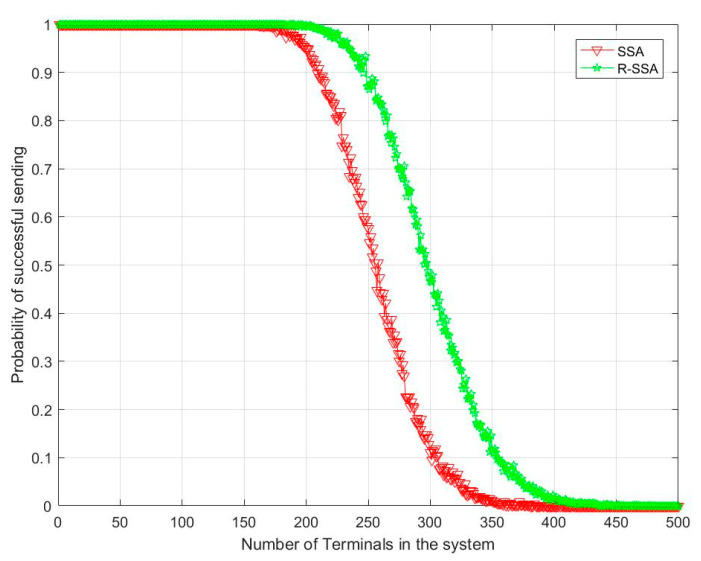
R-SSA vs. SSA in the probability of successful sending.

It can be seen from Figure 15 that after adding the collision backoff mechanism, the probability of successful terminal access increases. When the number of terminals is 250, there is also a probability of successful access of nearly 50%.

**Figure 16:**
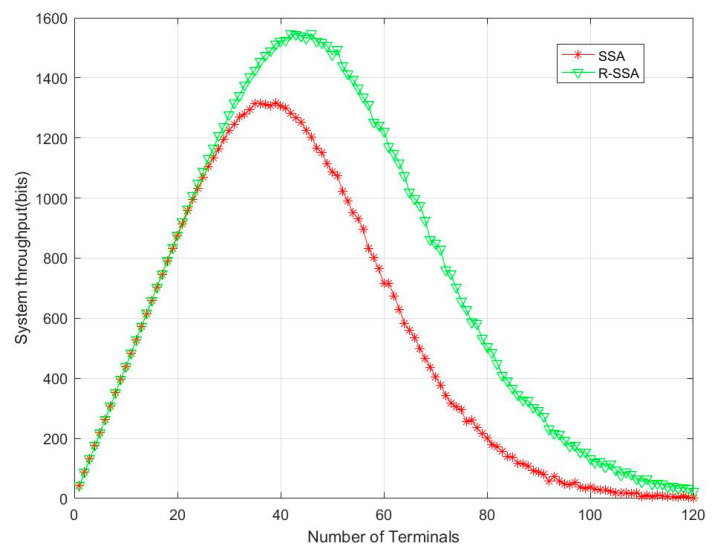
R-SSA vs. SSA in the throughput.

Figure 16 shows that the amount of effective data in the R-SSA system has also increased accordingly, and the effective data throughput in the system peaks when 48 terminals are connected.

## 4. Discussion

This paper compares the commonly used random access protocols. In the constructed low-orbit satellite beam-hopping IoT scenario, the SSA protocol is analyzed, simulated, and verified. At the same time, an improved SSA scheme, based on the collision backoff mechanism, is proposed.

From a theoretical point of view, the use of the SSA protocol in low-orbit satellite beam-hopping IoT scenarios is limited by the total number of terminals, the transmission frequency of a single terminal, and the parallel demodulation capability of the system. The increase in the total number of system terminals and the increase in the terminal transmission frequency set by the system will greatly improve the collision probability of the system. On the other hand, the increase in the number of parallel demodulations increases the terminal’s collision tolerance threshold, reduces the probability of real collision, and improves the probability of terminal transmission success from a practical point of view. From a theoretical point of view, the use of the SSA protocol in low-orbit satellite beam-hopping IoT scenarios is limited by the total number of users, the transmission frequency of a single terminal, and the parallel demodulation capability of the system. The increase in the total number of system terminals and the increase in the terminal transmission frequency set by the system will greatly improve the collision probability of the system. On the other hand, the increase in the number of parallel demodulations increases the user’s collision tolerance threshold, reduces the probability of real collision, and improves the probability of terminal transmission success from a practical point of view. From the simulation results, it can be seen that with the increase in the number of system terminals, the maximum throughput and stable operation range of the system increases. However, there are spikes in the maximum throughput of the system due to the parallel demodulation capability. The system performance drops sharply after exceeding the peaks. Under the same load, the parallel demodulation capability directly affects the probability of successful packet transmission. The simulation results show that, in the system design, it is necessary to estimate the maximum capacity of the system and determine the appropriate terminal transmission frequency according to the actual concurrent demodulation capacity.

In the R-SSA scenario that was improved in this article, since the arrival process of data packets in the system is the Poisson process, the retransmission process after the packet conflict is also the Poisson process. For a single collision window, the design of collision fallback leads to an increase in the probability that the user who previously collided randomly rewinds and falls back in the window. That is, the number of terminals that send data within the collision window increases. Although the probability of collisions within the window increases, because the SSA scheme is selected, as long as the two sets of data are not completely aligned with the chips, they can be received correctly. Therefore, the increase in the number of transmitting terminals in the collision window will not affect the normal access probability of the terminal, but can make more efficient use of the time slot resources and improve the probability of successful access.

## 5. Conclusions

In this paper, R-SSA, a spread-spectrum ALOHA protocol based on the collision backhaul mechanism, is proposed. The simulation results show that the use of this protocol in the multi-beam low-orbit satellite IoT communication system can improve the probability of the random access of terminals and increase the system’s tolerance for collisions. In the low-orbit satellite IoT scenario, realizing the random access of IoT terminals by combining beam-hopping with the R-SSA protocol described in this paper can improve the success rate of IoT terminal access and the effective data volume of the system, and improve the system’s tolerance for collision. However, it is necessary to further consider the simplified design of interactive protocols and the synchronization of the whole network of IoT terminals in practical applications.

## Figures and Tables

**Table 1:** Simulation parameters of CRDSA.

Parameters	Values
N_iter_	0, 1, 2, 3, 15
Data length(L)	256 bits

**Table 2:** Simulation parameters of SSA in the scenario of beam-hopping low-orbit satellite IoT.

Parameters	Values
Burst length (Tc)	250 ms
Data length (L)	256 bits
Number of concurrent demodulations	10~200
Number of system users	2000
Single-user sending frequency	1 s, 2 s, 5 s, 15 s, 25 s

**Table 3:** Simulation parameters of R-SSA in the scenario of beam-hopping low-orbit satellite IoT.

Parameters	Values
Burst length (Tc)	250 ms
window length(L)	256 bits
Number of concurrent demodulation(M)	10~200
Number of system users	120~2000
Expected number of terminals	6~20
Spread factor	31
Number of collision windows	2
Single-user sending frequency	1 s, 2 s, 5 s, 15 s, 25 s

## Data Availability

Not applicable.
